# The expression of substance P and calcitonin gene-related peptide is associated with the severity of tendon degeneration in lateral epicondylitis

**DOI:** 10.1186/s12891-021-04067-1

**Published:** 2021-02-21

**Authors:** Soo-Hong Han, Hyung Kyung Kim, Yoon Jang, Hyeon Hae Lee, Jeongbae Rhie, Donghun Han, Jongbeom Oh, Soonchul Lee

**Affiliations:** 1 Department of Orthopaedic Surgery, CHA Bundang Medical Center, CHA University School of Medicine, 335 Pangyo-ro, Bundang-gu, Gyeonggi-do, Seongnam-si, 13488 Republic of Korea; 2 Department of Pathology, Kyung Hee University Hospital at Gangdong, Kyung Hee University, College of Medicine, Seoul, Republic of Korea; 3 Department of Occupational and Environmental Medicine, Dankook University College of Medicine, Cheonan, Republic of Korea

**Keywords:** Substance P, Calcitonin gene-related peptide, Tendon degeneration, Lateral epicondylitis, Tendinopathy

## Abstract

**Background:**

In this study, we investigated whether substance P (SP) or calcitonin gene-related peptide (CGRP) expression is associated with tendon degeneration in patients with lateral epicondylitis.

**Methods:**

Twenty-nine patients who underwent surgical treatment for lateral epicondylitis were enrolled in the final analyses. Extensor carpi radialis brevis tendon origins were harvested for histological analysis.

**Results:**

SP and CGRP immunostaining were negative in healthy tendons but positive in degenerative tendons; moreover, their immunoreactivity increased with degeneration severity. Univariate analysis indicated that variables such as the preoperative visual analog scale (VAS) score or SP or CGRP expression levels were significantly associated with the Movin score. However, multivariate analysis revealed that only higher SP and/or CGRP signals were associated with higher Movin scores. Elevations in SP or CGRP expression were also linked with significantly severe preoperative VAS scores.

**Conclusion:**

We demonstrated that tendon degeneration severity is associated with increased SP and CGRP expression in the biopsy samples of lateral epicondylitis.

**Supplementary Information:**

The online version contains supplementary material available at 10.1186/s12891-021-04067-1.

## Background

Lateral epicondylitis, commonly known as tennis elbow, refers to a pathological condition with varying degrees of pain or point tenderness at the common extensor origin [1–3]. This condition has an annual estimated prevalence of 1–3% in the general population and is one of the most frequently diagnosed elbow conditions typically occurring after strenuous musculoskeletal movements or microtrauma incidents [4]. Since its first description in 1883, many authors have proposed various explanations such as micro-tears owing to repetitive loading, degenerative changes owing to abnormal vascularity, and oxygen free-radical injuries [5–8]. Nonetheless, the multifactorial nature of lateral epicondylitis hinders the understanding of its exact biochemical mechanism [9].

Histopathologically, lateral epicondylitis is characterized by angiofibroblastic proliferation with collagen tissue fibrillation and chronic inflammation. The presence of a disorganized extracellular matrix with increased angiogenesis, immature fibroblasts, and inflammatory cells can be observed in the disrupted tendon [10–16]. Nociceptive neuromediators act as primary components in pain pathophysiology. Multiple studies have specifically implicated the neuropeptides, Substance P (SP) and calcitonin gene-related peptide (CGRP), as potential tendinopathy-related factors [5, 17–20].

The expression of SP, CGRP, and their receptor, neurokinin-1, has been reported at the proximal extensor carpi radialis brevis (ECRB) tendon, the main pathological lesion in lateral epicondylitis [17, 21]. Peterson et al. used positron emission tomography and the neurokinin-1 specific radioligand to examine patients with lateral epicondylitis and found that the radioligand signal intensity was higher in the affected arm than in the unaffected arm [22]. Additionally, Fedorczyk et al. showed increased endogenous SP expression before tenocyte hypercellularity upon tendon loading [23]. The findings collectively indicate the importance of SP or CGRP in the pathogenesis of lateral epicondylitis [17–20].

However, to the best of our knowledge, no study has reported the relationship between SP or CGRP expression and the tendon degeneration level in lateral epicondylitis. Thus, we investigated the associations of SP and CGRP protein expression with the histological severity of tendon degeneration. We hypothesized that SP and CGRP expression would be associated with tendon degeneration severity in patients with lateral epicondylitis.

## Methods

### Design

This research was approved by CHA Bundang Medical Center, CHA University, Institutional Review Board (Registration no. 2013–01–174-012). All experiments were performed in accordance with the Declaration of Helsinki, and the protocol was approved by the institutional review board. Informed consent was obtained from all the participants. Clinical and histological data of the study participants from January 2010 to December 2015 were collected and analyzed.

### Study population

**Fig. 1:**
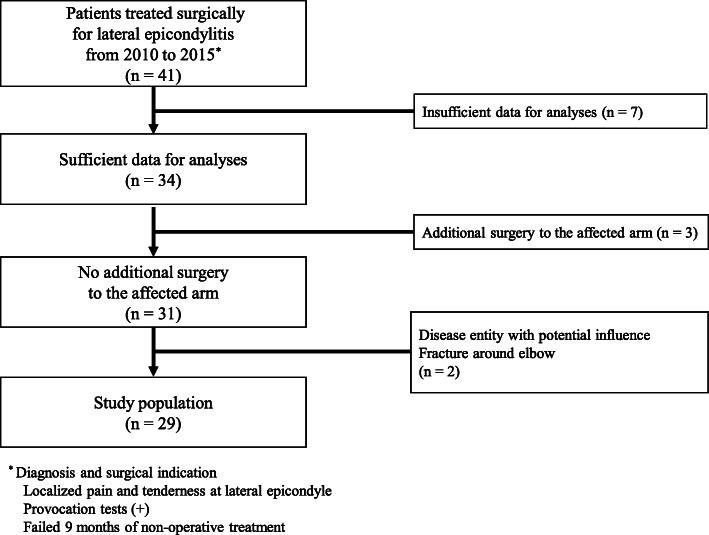
Flow chart illustrating the study population selection criteria

Only patients with a clinical diagnosis of lateral epicondylitis were included in this study. All patients had well-localized radioulnar joint pain and tenderness over the lateral epicondyle. Pain was also evoked by provocation tests, which included the following physical examinations: gripping resisted wrist extension with radial deviation or resisted third digit extension. Surgical treatment was performed on patients who had failed non-operative treatment for a minimum of 9 months, including the use of braces, physiotherapy (ultrasound, electrical stimulation, heat therapy, and manipulation), and non-steroidal anti-inflammatory medication, single/multiple platelet-rich plasma, or corticosteroid injection. We judged the failure of non-operative treatment at least 3 months after the last injection. A modified Nirschl procedure was performed on all patients by a single surgeon [24] and the patients were followed up for at least 2 years after surgery. Forty-one patients were originally identified; data from seven of these patients were insufficient for analysis and were excluded. Three patients with a history of previous surgery in the affected arm were excluded, along with two others who had pre-existing arm conditions such as rheumatoid arthritis or medial epicondylitis. Twenty-nine elbows (25 dominant arms and four non-dominant arms) in 29 patients (14 males and 15 females) were enrolled in the study (Fig. 1). The mean patient age was 48 years (range, 38–58 years). Eight patients were included in the control group (five males and three females). The mean age of the control group was 35 years (range, 22 to 47 years). Remains of hamstring (seven patients) or patellar tendon (one patient) from other patients undergoing anterior cruciate ligament reconstruction were used as healthy controls to histologically compare and validate the harvested specimens from patients with lateral epicondylitis [25].

### Preoperative and postoperative clinical data

The following clinical data were collected at the outpatient department for all patients: gender, age, job, a record of industrial accident compensation, dominant or non-dominant arm, smoking history, alcohol consumption, plain radiograph of the elbow during operation, body mass index, and preoperative symptom duration. In addition to the baseline patient data, postoperative symptom improvements, range of motion, and radiographic changes were also evaluated. Patients were obliged to complete two self-assessing questionnaires for pain severity and functional disability. The visual analog scale (VAS), one of the most sensitive pain rating scales, was utilized to determine pain on a scale from 0 to 10 [16]. The mean disability of the arm, shoulder, and hand (DASH) outcomes measured the functional defects at the upper extremity on a scale of 100, with a higher score indicating a greater level of disability [26, 27]. Both VAS and DASH scores were obtained before and 2 years after surgery. The measurements taken after 2 years were assumed to reflect the final clinical outcomes. The patient demographics and clinicopathological data are summarized in Table 1.

**Table 1:** Patient Demographics and Clinicopathological Data

Variables	No. (%) or Mean ± SD
Gender	
Male	14 (48.28)
Female	15 (51.72)
Age (years)	48.41 ± 10
Job^a^	
Housekeeping	4 (13.79)
White-collar	12 (41.38)
Blue-collar	13 (44.83)
Industrial accident compensation^a^	
No	27 (96.4)
Yes	2 (7.1)
Affected arm^a^	
Dominant	25 (86.21)
Non-dominant	4 (13.79)
Smoking^a^	
No	22 (85.86)
Yes	7 (24.13)
Alcohol consumption^a^	
None	20 (68.96)
Social	7 (24.13)
Heavy^c^	2 (6.9)
Plain radiograph at operation^a^	
Normal	13 (44.83)
Abnormal^d^	16 (55.17)
Body mass index (kg/m^2^)	23.74 ± 3.08
Preoperative	
Symptom duration (months)	22.31 ± 15.91
VAS score	4.62 ± 1.21
DASH score	58.31 ± 13.63
Postoperative^a^	
VAS score	0.9 ± 1.3
DASH score	15.1 ± 12.7
Postoperative range of motion^a, b^	
Full	27 (96.4)
Limited	2 (7.1)
Neuropeptide expression	
SP	2.05 ± 1.29
CGRP	5.11 ± 5.03

*SD* Standard deviation, *SP* Substance P, CGRP Calcitonin gene-related peptide, ^a^categorical variables, ^b^evaluated at two-year follow-up (two years after operation). ^c^indicates the patient who drinks on five or more days per month. ^§^includes sclerotic change of the lateral condyle and calcification of the extensor tendon origin

### Pathological review: evaluation of tendon degeneration

Biopsy specimens (approximately 1 cm^3^) were taken from ECRB tendon origins during surgery; they appeared dull, gray, friable, and edematous. Sampling was performed only after informed consent was obtained. Tendon degeneration in each harvested tissue was analyzed quantitatively. All specimens were fixed in 10% formalin, embedded in paraffin, and cut into 4-μm serial sections for H&E and Alcian blue staining. H&E staining was performed using an automated staining machine (Leica ST5010 Autostainer XL). Alcian blue (pH 2.5) staining was performed on deparaffinized sections according to the standard protocols: Alcian blue staining at room temperature for 30 min followed by nuclear fast red staining for 3 min. The Movin score was adopted to classify degrees of tendinopathy based on H&E and Alcian blue staining [28]. The 4-point scoring system comprised 0 (normal), 1 (mild), 2 (moderate), and 3 (severe), by including the following assessment criteria: fiber structure, fiber arrangement, size and shape of cell nuclei, collagen stainability, extracellular matrix, vessel density, hyalinization of stroma, and proteoglycan content (Supplemental Table 1) [29]. All samples were blindly examined by an orthopedic surgeon, pathologist, and a medical student after proper training.

### Pathological review: Immunohistochemical staining for SP and CGRP

Immunohistochemical staining for SP and CGRP was performed on lateral epicondylitis tendon specimens. All steps were performed following the manufacturer’s instructions, with minor modifications. Samples were sliced into 4 μm thick tissue sections from formalin-fixed, paraffin-embedded tissue blocks for immunohistochemical staining using the Bond Polymer Intense Detection system (Vision BioSystems, Victoria, Australia). After deparaffinization using Bond Dewax Solution (Vision BioSystems), antigen retrieval was performed for 30 min at 100 °C using Bond ER Solution (Vision BioSystems). Endogenous peroxidase activity was quenched for 5 min at room temperature. Then, the sections were incubated for 15 min at ambient temperature with mouse monoclonal antibodies against SP (1: 400, Abcam, Cambridge, UK) or CGRP (1: 200, Abcam, Cambridge, UK). A biotin-free polymeric horseradish peroxidase-linker antibody conjugate system was utilized to visualize the spatial and temporal localization of SP and CGRP. The sections were counterstained with hematoxylin for observation under a light microscope (Eclipse Ni-U, Nikon, Japan).

### Pathological review: quantitative evaluation of SP and CGRP protein expression

**Fig. 2:**
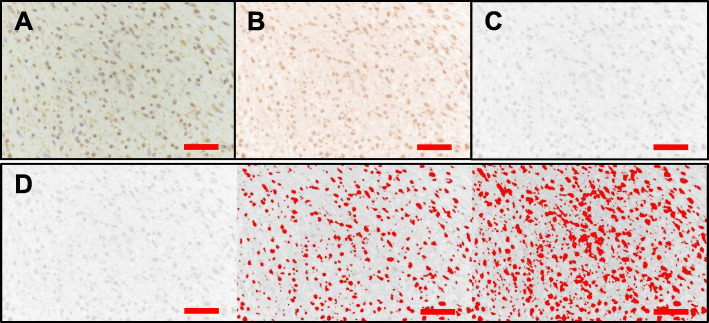
Quantitative image analyses of SP and CGRP expression. Quantitative analysis was achieved by counting the positive brown particles pixel-by-pixel in immunohistochemistry images using ImageJ (National Institute of Health, Bethesda, Maryland, USA). First, digital images of SP and CGRP immunostaining were obtained (**a**). Next, the color deconvolution plugin was used to isolate immunopositive particles into the brown image from the original image using the H-DAB vector (**b**). The positive particles of the brown image were converted into black and white images for recognition (**c**). Three montages of each image were generated for accurate measurements and threshold determination, and the positive signal area was measured in pixels (**d**). SP, substance P; CGRP, calcitonin gene-related peptide; magnification, × 400; scale bar = 50 μm

Digital images of SP and CGRP immunostained sections were randomly obtained from five areas of the degenerative region under 400x magnification. Each image was analyzed using the digital image analysis program, ImageJ (National Institute of Health, Bethesda, Maryland, USA). Quantitative analysis was performed by counting positive brown particles in immunohistochemistry pixel-by-pixel. The Color Deconvolution plugin and H-DAB vector enabled isolation of the brown image composed of positive particles from the original picture. The brown image was converted into a black-and-white photo so that the ImageJ program could detect the positive particles on a black-and-white scale. The focus of the field in each image may be uneven; thus, three montages were generated and the measurement threshold was determined to obtain more accurate values (Fig. 2) [30, 31]. The area fraction value was measured in ImageJ by dividing the pixels of the immunopositive area with the pixels of the total area. This value was used as the staining score. Additionally, for the normalization of protein expression, the number of tenocytes was measured semi-manually using ImageJ. The cell count jar plugin in ImageJ enables direct marking of a cell image. Every tenocyte in each image was marked manually, and the program measured the Loser total cell number in each marked image.

### Statistical analysis

We determined the overall summary statistics for continuous and categorical variables in terms of means and standard deviations, and frequencies and percentages, respectively. Intra-observer and inter-observer reliability of histologic evaluation were determined using intraclass correlation coefficients based on the 95% confidence interval for absolute agreement. A correlation coefficient of 0–0.2 indicated poor reliability; 0.21 0.4 was fair, 0.41. 0.6 was moderate, 0.61. 0.8 was good, and greater than 0.81 indicated excellent reliability. After the correlation analyses of independent variables using Pearson’s correlation test, univariate analyses were performed to evaluate associations between variables and tendon degeneration (shown as Movin score). Linear regression models were applied for continuous variables, whereas analyses of variance and t-tests were used for categorical variables. After univariate analyses, various contributing factors including SP, CGRP expression, and several other possible confounders such as sex, age, job, presence of medical compensation, past medical history, invasion of the dominant/non-dominant arm, preoperative symptom duration, preoperative plain radiograph of the elbow, range of motion of the arm, body mass index, smoking history, and alcohol consumption were entered into a stepwise multiple regression model to investigate their individual and combined effects on tendon degeneration. Final variables were selected based on the model suitability scales such as adjusted R square, *Akaike* information criterion, and Mallow’s Cp. Multicollinearity was tested using the variance-inflation factors function in R (R Foundation for Statistical Computing, Vienna, Austria), and multiple linear models were verified using the Olsrr package. All statistical analyses were performed using the statistical software, R. The statistical significance threshold was set at *p* <  0.05 (Bonferroni correction was performed to account for multiple tests).

## Results

No complications such as stiffness or instability of the elbow, nerve injury, or infection occurred after surgery. Overall, pain and elbow function scores improved in all patients after the operation. Visual analog scale (VAS) scores declined significantly from a preoperative value of 4.62 (range, 3–7) to a two-year follow-up value of 1.06 (range, 0–3) (*p* <  0.05). The DASH score decreased from 57.23 (range, 30–83.3) before surgery to 17.82 (range, 0–42) at the two-year follow-up (*p* < 0.05).

**Fig. 3:**
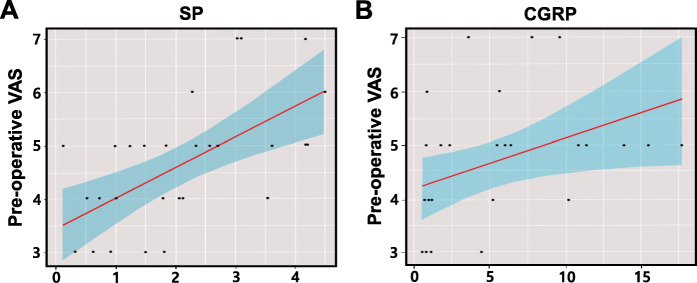
Correlations between SP or CGRP expression and preoperative VAS scores. There were significant positive correlations between SP (**a**) and CGRP (**b**) expression and preoperative VAS scores (SP: *p* < 0.05, *r* = 0.61, CGRP: *p* < 0.05, *r* = 0.39). VAS, visual analog scale; SP, substance P; CGRP, calcitonin gene-related peptide. The score on the x-axis shows the staining score, which was calculated by dividing the pixels of the immunopositive area with the pixels of the total area

Correlation tests between each parameter indicated that the postoperative VAS score and postoperative DASH score showed strong correlations (*r* = 0.85, *p* < 0.001); therefore, only postoperative DASH scores were used in the following analyses (Supplemental Fig. 1). Additionally, univariate analysis results revealed that SP and CGRP expression were significantly related to the preoperative VAS score (r = 0.61, *p* = 0.0006 for SP expression and r = 0.39, *p* = 0.034 for CGRP expression) (Fig. 3). This suggests that patients with higher SP and CGRP expression are more likely to experience severe pain before surgery. However, the correlation test results suggested that postoperative improvement (postoperative DASH score) was not related to SP or CGRP levels (r = 0.14, *p* = 0.36 for SP and r = 0.13, *p* = 0.23 for CGRP).

### Tendons with severe histological degeneration had concomitantly high SP and CGRP protein expression

For the histological analyses, the inter-observer and intra-observer reliabilities were 0.79 (good) and 0.83 (excellent), respectively. Tendons harvested from patients with lateral epicondylitis were validated by comparison with a healthy control group. All tendons from lateral epicondylitis showed characteristic tendon degeneration features at varying degrees. Samples presented disorganized tendon fibers with higher cellularity and vessel formation in hematoxylin and eosin (H&E) staining. In Alcian blue staining, the degenerative tendons showed marked amounts of ground substances consisting of proteoglycans and glycosaminoglycan with several mucoid patches and vacuoles between fibers. They also showed exceedingly increased SP and CGRP immunoreactive area fractions compared to the healthy control group.

**Fig. 4:**
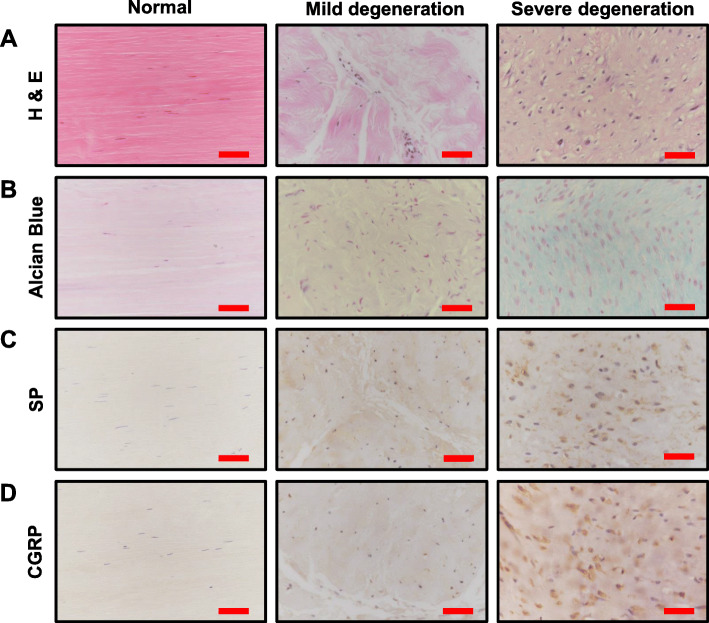
Representative histologic images of healthy control and degenerated tendons. H&E staining (**a**) of healthy tendons displayed arrays of straight, compact fibers with linear shaped interfascicular inconspicuous nuclei. As degeneration progressed, the fibers started to lose polarity and became thinner and wavy. Tenocytes increased in number and size, and their nuclei changed into ovoid shape. Alcian blue staining (**b**) of degenerated tendon tissues showed interfascicular ground substances and acid mucopolysaccharides, which are rarely observed in healthy tendons. SP (**c**) and CGRP (**d**) immunoreactions were elevated in proportion with the degree of degenerative changes, but healthy tendons showed negative reactions to immunohistochemical staining. Values below the mean Movin score of 17.5 were defined as mild degeneration. Values above the mean Movin score were defined as severe degeneration. SP, substance P; CGRP, calcitonin gene-related peptide; magnification × 400; scale bar = 50 μm

Next, the staining scores for SP and CGRP were determined quantitatively using immunohistochemistry images. The pixels with positive brown particles were counted using ImageJ (Fig. 2). Mean staining scores in specimens were found to be 2.05 for SP and 5.11 for CGRP. In contrast, healthy controls were negative for SP and CGRP immunohistochemical staining of tenocytes (Fig. 4). Next, the total expression of SP and CGRP was normalized to the cell number because the degenerated tendons had more cells. The results demonstrated that tendon samples with a higher Movin score had significantly higher SP and CGRP expression per cell (Supplemental Fig. 2).

### Higher SP and/or CGRP expression was associated with greater tendon degeneration (Movin score)

**Fig. 5:**
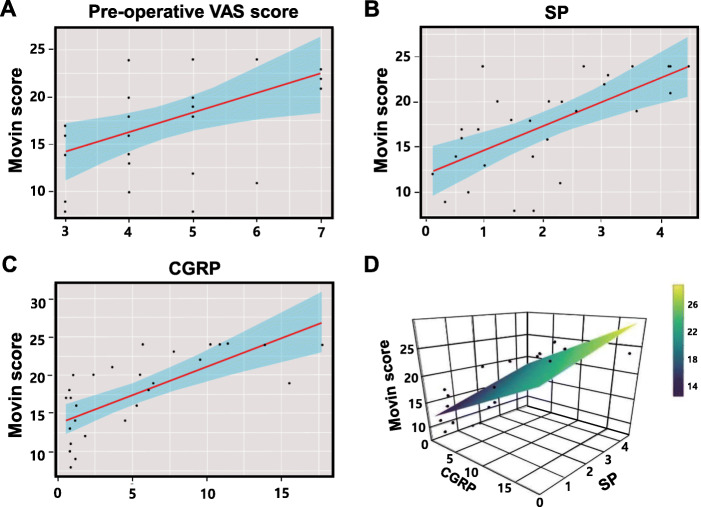
Correlation of SP and/or CGRP expression with tendon degeneration (Movin score). The association between various variables and tendon degeneration severity (Movin score) was analyzed using the linear regression test. The Movin score was significantly associated with the parameters including preoperative VAS score (**a**), SP (**b**), and CGRP (**c**) expression levels (*r* = 0.22, p < 0.05; *r* = 0.43, *p* < 0.001; and *r* = 0.50, *p* < 0.001 respectively). The association of both SP and CGRP with the Movin score was visualized by a three-dimensional graph (**d**). VAS, visual analog scale; SP, substance P; CGRP, calcitonin gene-related peptide

Univariate analyses demonstrated that preoperative VAS score (*r* = 0.22, *p* < 0.05), as well as SP (*r* = 0.43, *p* < 0.001) and CGRP (*r* = 0.50, p < 0.001) expression levels were significantly associated with higher Movin scores (Fig. 5). Multiple regression models were generated to determine the parameters influencing the Movin score. SP and CGRP expression scores were selected as associated parameters in stepwise regression, indicating that SP and CGRP expression were highly associated with the extent of tendon degeneration. The results indicated that the prediction model including SP and CGRP accounted for 52.63% of the Movin score. Preoperative VAS scores were excluded from the analysis because they did not statistically contribute to the model in the adjusted R square, *Akaike* information criterion, and Mallow’s Cp (Supplemental Fig. 3). Collectively, the equation was: Prob (Tendon degeneration, Movin score) = 1.38. × SP expression + 0.61 × CGRP expression + 11.90. Table 2 summarizes the results of the multiple regression models.

**Table 2:** Full Model from Stepwise Multiple Linear Regression Analyses

Dependent variable	Independent variables	Coefficient	95% CI	Adjusted r^**2**^	***p***-value
**Movin score**				0.60	< 0.05
	Intercept	11.90	10.7–13.1		
	SP	1.38	0.73–2.04		
	CGRP	0.61	0.43–0.80		

*CI* Confidence interval, *SP* Substance P, *CGRP* Calcitonin gene-related peptide

## Discussion

Disruption of neuronal regulation, regulated by the neuromediators SP and CGRP, is likely to contribute to tendon degeneration [32]. We hypothesized that either SP or CGRP protein levels would be closely linked to tendon degeneration. Histopathologically quantified data supported this hypothesis by revealing significant associations between the degrees of tendinopathy and the SP and CGRP levels.

Infection or injury is known to trigger neuropeptide release, which can induce an inflammatory reaction [33]. However, the effect of neuropeptides like SP or CGRP on tendinopathy development or acceleration is debatable. Ljung et al. compared SP and CGRP immunoreactivity between patients with lateral epicondylitis and healthy volunteers, and concluded that neurogenic inflammation may be implicated in the etiology of lateral epicondylitis [21]. Likewise, Uchio et al. demonstrated how neuropeptides (SP and CGRP) and cytokines (interleukin-1α and tumor growth factor-β) contribute to lateral epicondylitis progression [6]. However, these studies were only conducted on small sampling scales of six and nine patients, respectively.

Similarly, SP and CGRP levels are reported to be increased in tendon fibroblasts in animal models of collagenase-induced tendinopathy [34]. In this context, SP and CGRP have been highlighted as critical factors in chronic tendon pain and thickening. Gene expression analyses performed by Backman et al. illustrated how mechanical loading on tendon cell cultures significantly increased SP and neurokinin-1 receptor mRNA levels. They also observed increased endogenous SP production in an animal model [35, 36]. Later, the same group recapitulated these changes, including hypercellularity and angiogenesis, by injecting SP close to the tendons in vivo [37]. Zhou et al. also discussed the effects of SP on pluripotent tendon cell proliferation and differentiation in vivo and in vitro in rat patellar tendon injection models [38]. They showed that SP upregulated the expression of non-tenocyte genes but downregulated the expression of tenocyte-related genes during pluripotent stem cell differentiation. Histological examination revealed that SP induced in vivo tissue disorganization, which later developed into tendinopathy.

On the contrary, other studies have reported favorable roles of SP and CGRP in tendon healing processes. Carlsson et al. argued that SP enhances tissue recovery via multiple modalities such as inflammatory response, pain transmission, cellular proliferation, and increased vascular permeability in acute tendon injury or rupture [39]. Fong et al. discovered that cyclic tensile loading of tenocyte cultures along with the administration of exogenous SP, triggers collagen remodeling through its synergistic enhancement of matrix metalloproteinase 3 [32]. Another in vivo study by Steyaert et al. highlighted improvements in tendon recovery rates by injecting SP into the paratendinous region of an acute Achilles tendon injury [40].

In another aspect, Zhou et al. studied the effects of different SP concentrations on in vitro and in vivo tendon-derived stem cells [41]. Low dose SP (0.5 nmol) enhanced tenogenesis by inducing tenocyte-related genes, whereas high-dose SP (5.0 nmol) promoted tendinopathy-like changes in the tendon around the patella by activating non-tenocyte-related genes such as peroxisome proliferator activator receptor γ and collagen type II. Collectively, we can speculate that SP and CGRP have different effects on tendons according to their dosage and duration of exposure.

In this study, we discovered that preoperative VAS scores were significantly related to the SP and CGRP levels. We enlarged the sampling pool by collecting 29 tissue samples for 6 years, with at least a two-year follow-up. These results are consistent with those of previous studies; SP and CGRP are well-known causes of pain, hyperalgesia, vasodilation, and plasma extravasation [42, 43]. Interestingly, postoperative DASH and VAS scores improved in most cases regardless of SP and CGRP levels in the harvested tissue. Therefore, further research related to this result is warranted.

There are several limitations in our study. Although we demonstrated that SP and CGRP expression was significantly related to the degree of tendinopathy, it is unknown whether there is a causal relationship between neuropeptides and tendinopathy. Second, we managed to diagnose lateral epicondylitis in only clinical instances without the use of imaging studies such as magnetic resonance imaging (MRI). MRI in addition to pathological evaluation could have been helpful in diagnosing and grading the degrees of lateral epicondylitis. Third, statistical bias may have occurred because the expression patterns of healthy control and degenerated tendons were not compared under the same conditions. Hamstring or patella tendons were harvested as controls, which are likely to have been exposed to different physical loading conditions in daily activity. In addition, the healthy controls were much younger on average compared to the patients with lateral epicondylitis. Further, only the relative expression levels of SP and CGRP were compared because there are no known standard levels of SP and CGRP expression in healthy and degenerated tendons.

## Conclusions

Our data highlight that both SP and CGRP expression were increased in the tendon samples of lateral epicondylitis and that their expression levels are coupled with the degree of tendon degeneration. Additionally, these nociceptive neuromediators are associated with preoperative pain in lateral epicondylitis, suggesting that SP and CGRP might be related to the development of tendinopathy. Further studies are, therefore, necessary to develop novel therapeutics targeting SP and CGRP for treating tendon degeneration.

## Supplementary Information

**Additional file 1: Supplemental Figure 1.** Results of correlation tests between two variables: Correlations matrix. A: Age (years), B: Preoperative symptom duration, C: Preoperative VAS score, D: Preoperative DASH score, E: Postoperative DASH score, F: Postoperative VAS score, G: Body mass index, H: Movin score, I: SP expression, J: CGRP expression, Red box: Significantly correlated values. Values in each square box imply a correlation coefficient (*r*). VAS, visual analog scale; DASH, disability of arm, shoulder, and hand; SP, substance P; CGRP, calcitonin gene-related peptide.

**Additional file 2: Supplemental Figure 2.** Normalized SP and CGRP expression per cell. To calculate the SP and CGRP expression per cell, the cell numbers in five representative images were counted and the total SP and CGRP expression was normalized by cell number. The tendon sample with the higher Movin score (22 ≤ score ≤ 24) was determined to have significantly higher SP and CGRP expression per cell. Data are expressed as mean *±* standard deviation. ^*^ indicates a *p* value less than 0.05. SP, substance P; CGRP, calcitonin gene-related peptide.

**Additional file 3: Supplemental Figure 3.** Regression model accuracy. Regression model accuracy was tested using the adjusted R square (A), *Akaike* information criterion (B), and Mallow’s Cp (C)*.* Models excluding the preoperative VAS score showed better fit in all tests. VAS: visual analog scale.

**Additional file 4: Supplemental Table 1**. Movin score.

## Data Availability

The datasets used and/or analyzed during the current study are available from the corresponding author upon reasonable request.

## Abbreviations

SP: Substance P
CGRP: Calcitonin gene-related peptide
VAS: Expression, visual analog scale
DASH: Disability of the Arm, Shoulder, and Hand
MRI: Magnetic resonance imaging
