# Supplementary figures and images for: The expression of substance P and calcitonin gene-related peptide is associated with the severity of tendon degeneration in lateral epicondylitis
Source: BMC Musculoskelet Disord. 2021 Feb 21;22:210. doi: 10.1186/s12891-021-04067-1 (PMC7898744; doi:10.1186/s12891-021-04067-1)

Supplemental Fig. 1.

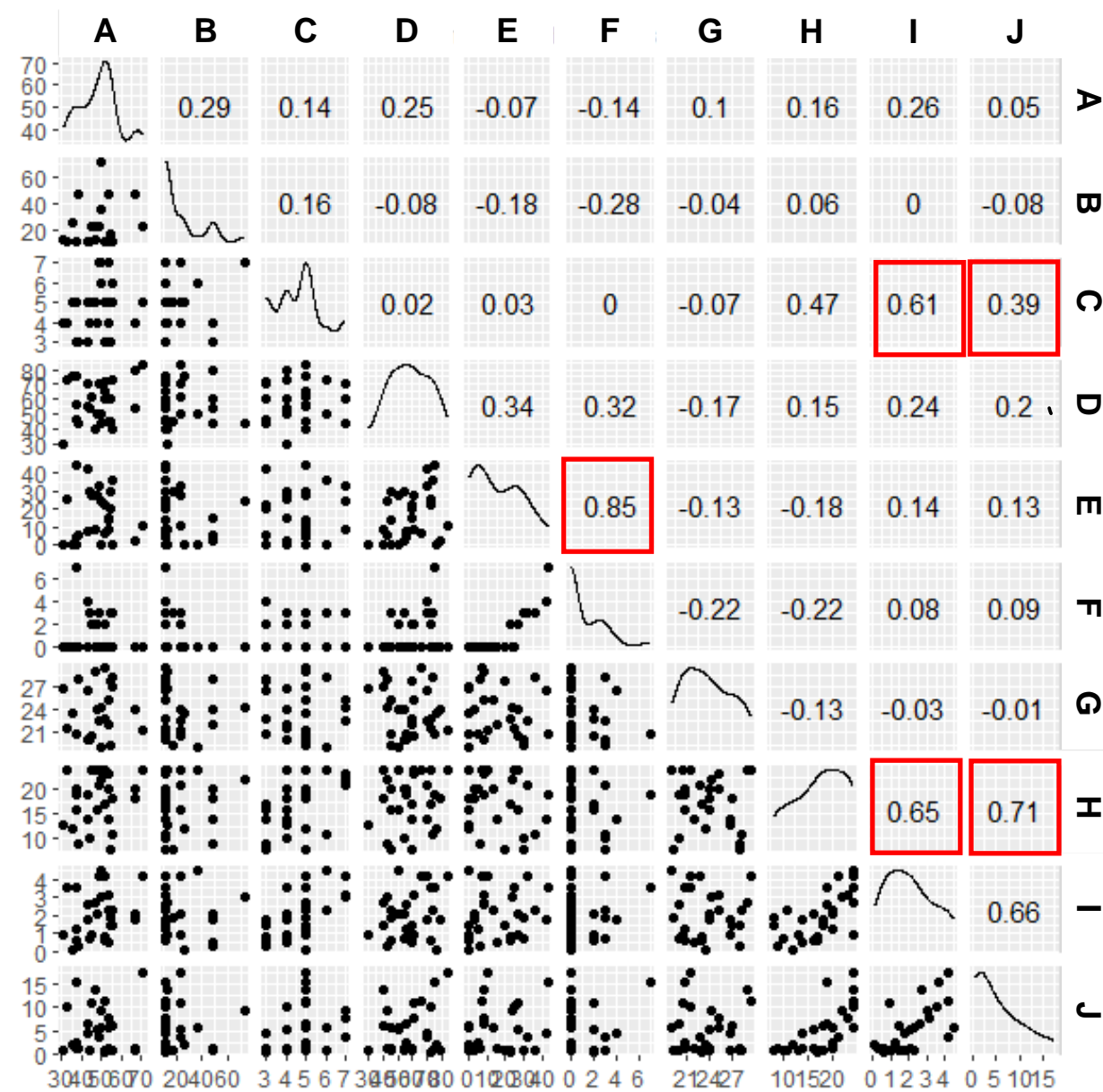

Supplement: Supplementary file 1 — Additional file 1: Supplemental Figure 1. Results of correlation tests between two variables: Correlations matrix. A: Age (years), B: Preoperative symptom duration, C: Preoperative VAS score, D: Preoperative DASH score, E: Postoperative DASH score, F: Postoperative VAS score, G: Body mass index, H: Movin score, I: SP expression, J: CGRP expression, Red box: Significantly correlated values. Values in each square box imply a correlation coefficient (r). VAS, visual analog scale; DASH, disability of arm, shoulder, and hand; SP, substance P; CGRP, calcitonin gene-related peptide. [file 12891_2021_4067_MOESM1_ESM.pdf]

Supplemental Fig. 2

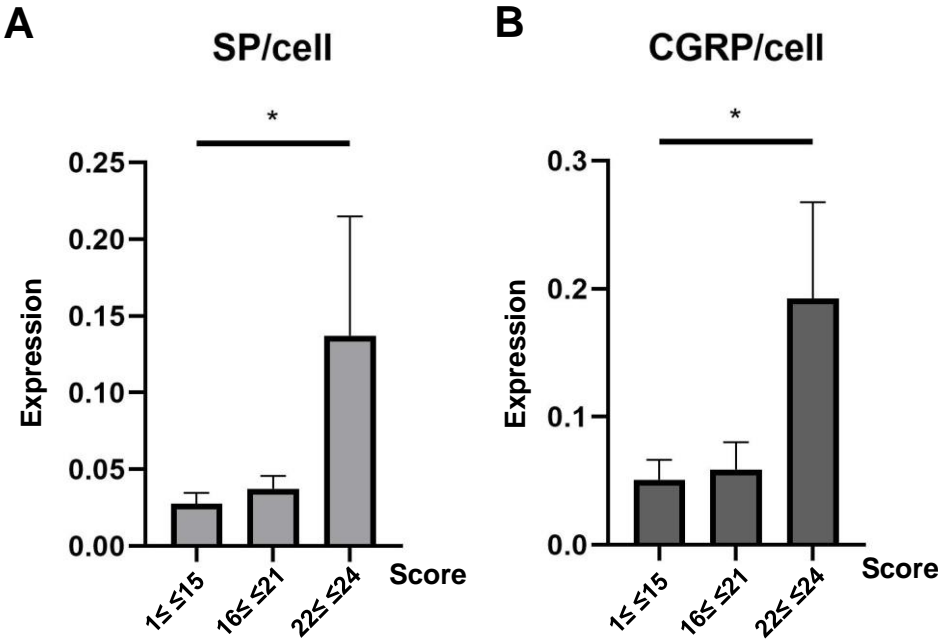

Supplement: Supplementary file 2 — Additional file 2: Supplemental Figure 2. Normalized SP and CGRP expression per cell. To calculate the SP and CGRP expression per cell, the cell numbers in five representative images were counted and the total SP and CGRP expression was normalized by cell number. The tendon sample with the higher Movin score (22 ≤ score ≤ 24) was determined to have significantly higher SP and CGRP expression per cell. Data are expressed as mean ± standard deviation. * indicates a p value less than 0.05. SP, substance P; CGRP, calcitonin gene-related peptide. [file 12891_2021_4067_MOESM2_ESM.pdf]

Supplemental Fig. 3. Regression model accuracy

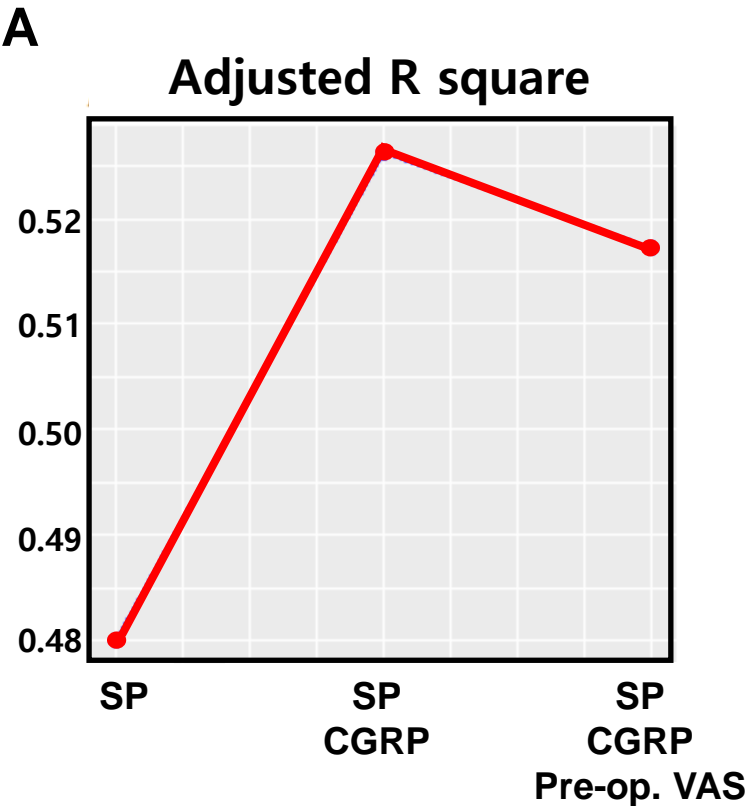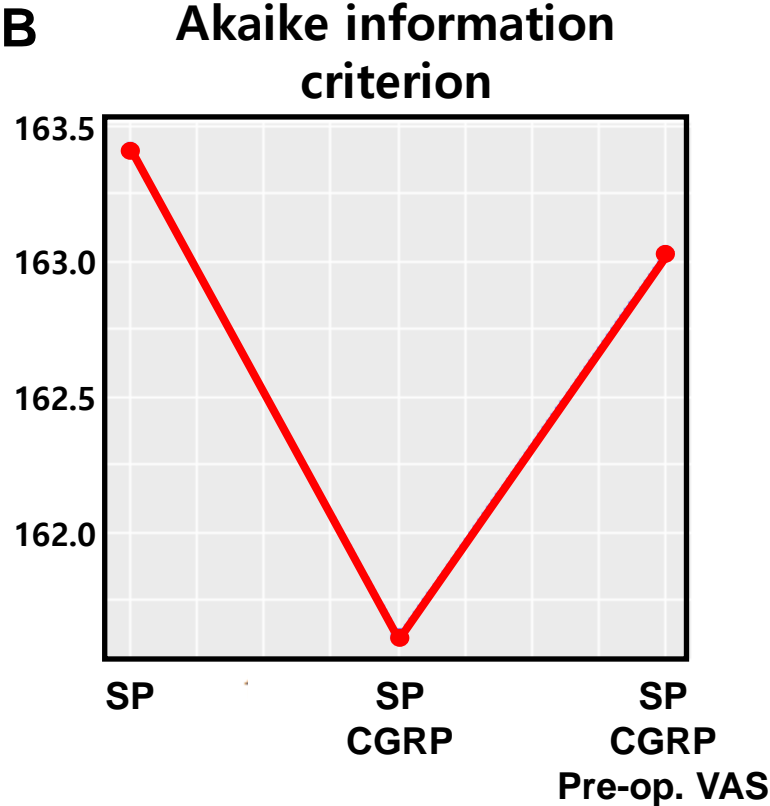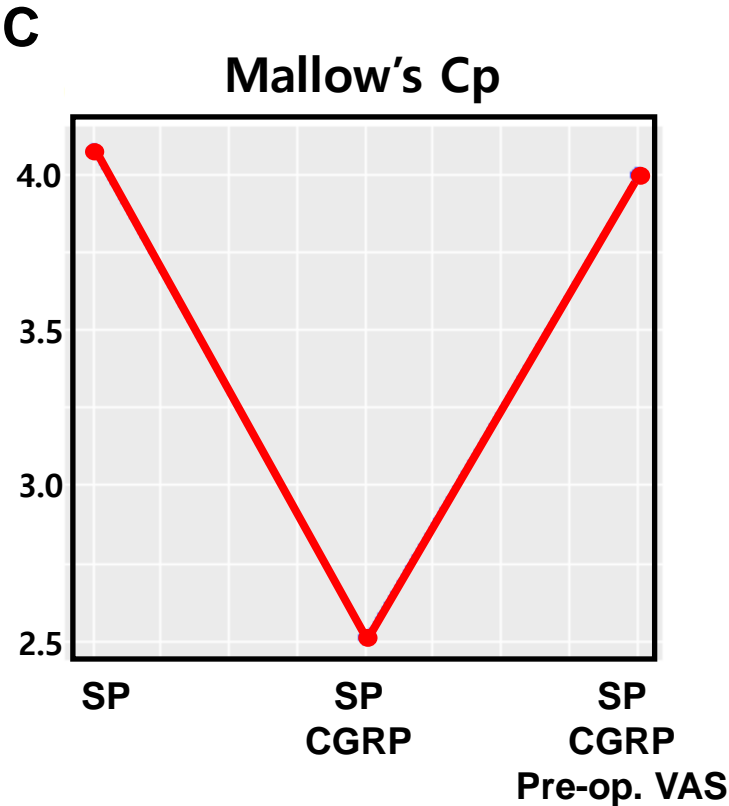

Supplement: Supplementary file 3 — Additional file 3: Supplemental Figure 3. Regression model accuracy. Regression model accuracy was tested using the adjusted R square (A), Akaike information criterion (B), and Mallow’s Cp (C). Models excluding the preoperative VAS score showed better fit in all tests. VAS: visual analog scale. [file 12891_2021_4067_MOESM3_ESM.pdf]
