# Supplementary material for: The expression of substance P and calcitonin gene-related peptide is associated with the severity of tendon degeneration in lateral epicondylitis
Source: BMC Musculoskelet Disord. 2021 Feb 21;22:210. doi: 10.1186/s12891-021-04067-1 (PMC7898744; doi:10.1186/s12891-021-04067-1)
Supplement: Supplementary file 4 — Additional file 4: Supplemental Table 1. Movin score. [file 12891_2021_4067_MOESM4_ESM.docx]

**Supplemental Table 1. Movin score**

| **Variables** | **Grade** | | | | |
| --- | --- | --- | --- | --- | --- |
|  | **0**  **(Normal)** | **1**  **(Slightly abnormal)** | **2**  **(Abnormal)** | **3**  **(Markedly abnormal)** |  |
| **Fiber structure** | Continue, long fiber | Slightly fragmented | Moderately fragmented | Severely fragmented |  |
| **Fiber arrangement** | Compacted and parallel | Slightly loose and wavy | Moderately loose, wavy  and cross to each other | No identifiable pattern |  |
| **Rounding of the nuclei** | Long spindle shape cells | Slightly rounding | Moderately rounding | Severely rounding |  |
| **Regional variations in cellularity** | Normal pattern | Slightly increased | Moderately increased | Severely increased |  |
| **Increased vascularity** | < 10% | 10–20% | 20–30% | > 30% |  |
| **Decreased collagen stainability** | < 10% | 10–20% | 20–30% | > 30% |  |
| **Hyalinization** | < 10% | 10–20% | 20–30% | > 30% |  |
| **Glycosaminoglycan content** | < 10% | 10–20% | 20–30% | > 30% |  |
